# Practice of Breastfeeding and its Barriers among Women Working in Tertiary Level Hospitals

**DOI:** 10.31729/jnma.4035

**Published:** 2019-02-28

**Authors:** Mukta Singh Bhandari, Pratibha Manandhar, Dipesh Tamrakar

**Affiliations:** 1 Department of Community Medicine, Kathmandu University School of Medical Sciences, Dhulikhel, Kavrepalanchok, Nepal; 2 Department of Community Medicine, Kathmandu Medical College, Duwakot, Bhaktapur, Nepal

**Keywords:** *breastfeeding*, *complementary feeding*, *working women*

## Abstract

**Introduction:**

Breastfeeding provides ideal food for newborns and prevents recurrent infection and malnutrition in infants. In Nepal, breastfeeding is a universal practice but exclusive breastfeeding is low. As there is increased involvement of women in workforce, practice of breastfeeding may have been compromised. The main objective of the study was to examine the practice and barriers of breastfeeding among women working in tertiary level hospitals of Nepal.

**Methods:**

This was a hospital based descriptive cross-sectional study. Study participants were women working in Dhulikhel Hospital and Kathmandu Medical College and Teaching Hospital and was conducted from December 2017 to June 2018. Ethical approval was taken from institutional review committee of both institutions. Total of 208 women were included in the study and face to face interview was conducted. Data entry and analysis was done using statistical package of social sciences.

**Results:**

Breastfeeding practice was universal and colostrum was fed by 195 (94%) women. Pre-lacteal feeding was given by 14 (7%) women and 119 (57%) initiated breastfeeding within one hour of birth. Total of 93 (45%) children were exclusively breastfed for any duration while 10 (11%) were exclusively breastfed for six months. The mean duration of breastfeeding was 14.57 months and 90 (43%) started complementary feeding before six months. Total of 97 (51%) women and 42 (47%) women stated work as barrier for not exclusively breastfeeding and early complementary feeding respectively.

**Conclusions:**

Exclusive breastfeeding was very low and children were breastfed for less than two years. Complementary feeding was also started earlier and work was stated as the main barrier for poor breastfeeding practice.

## INTRODUCTION

Breastfeeding provides adequate nutrition for growth and development of the infant. Thus, World Health Organization (WHO) recommends exclusive breastfeeding for the first six months of life which should be continued for up to two years.^1^ Infants who are not exclusively breastfed are more likely to develop infections and have increased mortality risk.^2^ Breastfeeding also has longer-term health benefits for the child like reducing the risk of overweight and obesity. In mothers, it reduces the risk of ovarian and breast cancer.^3^

In the context of Nepal, breastfeeding is a universal practice^4–6^ but only 66% of children are exclusively breastfed.^7^ Practice of breastfeeding among working women of Nepal is less known but studies done in other countries show work and lack of breastfeeding facilities as the main barrier for breastfeeding.^8–10^

Thus, this study aims to find out the practice and barriers of breastfeeding among women working at tertiary level hospitals of Nepal.

## METHODS

This was a descriptive cross-sectional study done at Dhulikhel Hospital (DH) and Kathmandu Medical College and Teaching Hospital (KMCTH) of Kathmandu University, both of which are tertiary level teaching hospitals.

Ethical approval was taken from Institutional Review Committee of Dhulikhel Hospital (Reference number 123/17) and Kathmandu Medical College and Teaching Hospital (Reference number 100120181). Verbal consent was taken from each respondent before the interview. The duration of study was from December 2017 to June 2018.

**Inclusion criteria:**

1. Currently working women, under the age of 45 with a child below five years of age.
2. Both lactating and non-lactating women.

**Exclusion Criteria:**

1. Women who lost their child.
2. Women who were sick.
3. Women who couldn't breastfeed due to medical condition.

The sample size was calculated using the given formula:


$$\begin{array}{l} \text{n}={\underline{\text{Z}}}^{\underline{2}}\underline{\times \text{p(1-p)}}\\ {\text{e}}^{2}\\ ={\text{(1}.96)}^{2}\times 0.159\times 0.841)/{\left(0.05\right)}^{2}\\ \text{=}205.3 \end{array}$$

where, n is the expected sample size

Z is the value at 95% confidence level (i.e. 1.96)
p is the estimated prevalence of total births in that area= 15.9%“
q is the probability of non-occurrence of p (i.e. 1-p) and
e is the estimated error (5% or 0.05)

It gives a sample size of 205.3 which was rounded to 206. Total of 103 each eligible women were planned to be taken from both hospitals.

A list of all eligible women including those working in academics, hospital, administration, account, pharmacy, hygiene was prepared which found 106 eligible women at DH and 110 at KMCTH with total of 216 women. As the sampling frame was small, all eligible women were approached for the study. Out of 216 women, total 103 eligible women from DH and 105 from KMCTH consented to participate in the study.

Data was collected using a semi-structured questionnaire by face to face interview method. The questionnaire included information about socio-demographic profile, natality history, practice of breastfeeding, complementary feeding, information about maternity related facilities and paid maternity leave.

Exclusive breastfeeding was defined as feeding of mother's milk including feeding of any medicine prescribed by the physician. Infants who were given pre-lacteal feeding and any other drink other than breast milk were excluded.

Pre-lacteal feeding was defined as giving of honey, ghee or water before giving breast milk to the newborn and which has been traditionally followed.

Complementary feeding was defined as any solid or semi-solid food given to the infant irrespective of breastfeeding practice.

A descriptive analysis of socio-demographic variables was done using mean, frequency, percentage and standard deviation. Data entry and analysis was done in Statistical Package for Social Sciences (SPSS) version 20.

## RESULTS

Total of 208 women were included in the study. The mean age of women was 30.33±3.74 years. Out of 208 women, 189 (91%) women followed Hindu religion. One hundred and forty two (68%) women had one child and 29 (14%) children were <age of six months. Total of 122 (58%) women had education of bachelor's degree and above, 105 (51%) women were involved in clinical work and 134 (64%) women worked full time (Table 1).

**Table 1.** Socio-demographic profile of working women.

Socio-demographic variables		n (%)
Age of mother	<25 years	22 (11)
	25-35 years	167 (80)
	35-45 years	19 (9)
	<6 months	29 (14)
	6 to 12 months	33 (16)
Age of the child	12 months to 24 months	61 (29)
	24 months and above	85 (41)
Parity	Primipara	142 (68)
	Multipara	66 (32)
	Chhetri	49 (24)
	Bramhin	73 (35)
Caste	Newar	67 (32)
	Tamang	9 (4)
	Others	10 (5)
	Hindu	189 (91)
Religion	Buddhist	12 (6)
	Christian	7 (3)
	Up to SLC	14 (7)
Education of woman	+2 and equivalent	72 (35)
	Bachelors	61 (29)
	Masters and above	61 (29)
Type of Family	Nuclear	77 (37)
	Joint	63 (63)
Type of delivery	Normal	61 (29)
	Cesarean section	147 (71)
	Clinical	105 (51)
	Academic	20 (10)
Type of work	Both clinical and academic	38 (18)
	Administrative	16 (8)
	Hygiene	19 (9)
	Technical work and assistance	8 (4)
Type of employment	Full time	134 (64)
	Shift	74 (36)

Breastfeeding practice was done by all 208 (100%) women and colostrum was fed by 195 (94%) women. Pre-lacteal feed was given by 14 (7%) mothers and the most common pre-lacteal feed was honey 7 (50%). Total of 119 (57%) the women initiated breastfeeding within one hour of birth. The mean duration of breastfeeding was 14.57±9.51 months (Mean±SD).

**Figure 1.**
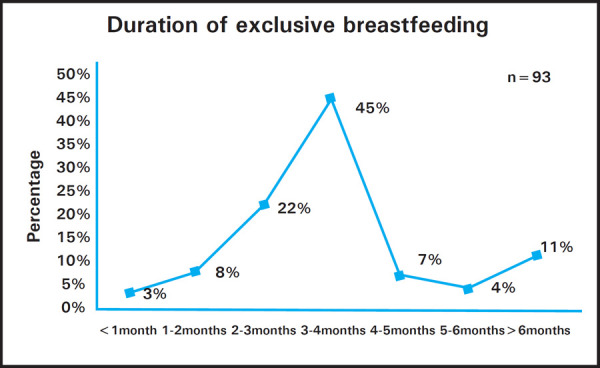
Duration of exclusive breastfeeding.

It was found that out of 208 women, 93 (45%) women breastfed their child exclusively for any duration and among the 93 women, only ten (11%) women breastfed exclusively for greater than six months (Figure 1). The mean duration of exclusive breastfeeding was 89.33±42.8 days (Mean±SD).

**Figure 2.**
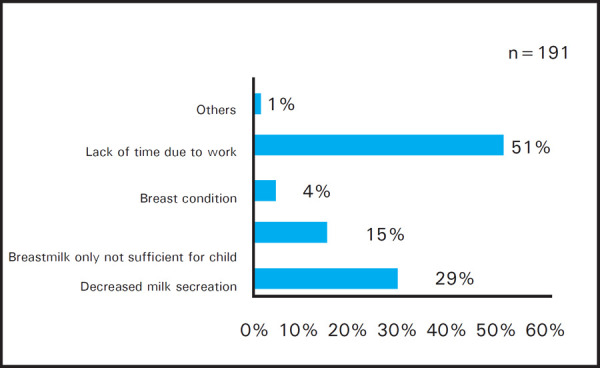
Barriers of exclusive breastfeeding.

Out of 208 women, 10 women who practiced recommended directions during both hospital stay and at home and seven women who practiced recommended directions at home only were excluded and barriers of exclusive breastfeeding were asked to 191 women who didn't practice exclusive breastfeeding as advised. Lack of time due to work was stated by 97 (51%) women as the most common barrier of exclusive breastfeeding and 2 (1%) women stated that poor latching and sickness of mother were other barriers (Figure 2).

Out of 208 children, complementary food was introduced before six months of age by 90 (43%) women. Out of 90 women, homemade lito or porridge was given by 46 (51%) women, 17 (19%) women gave mixed food, 16 (18%) women gave cerelac and 11 (12%) women gave rice and lentil as complementary food before six months of age. Mixed food included combination of cerelac, porridge and rice and lentil.

**Figure 3.**
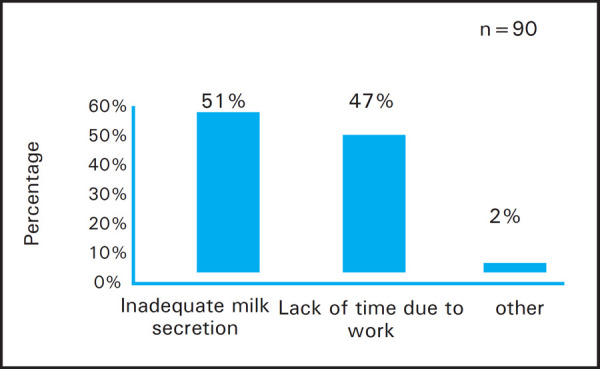
Reasons for starting complementary food early.

Total of 46 (51%) women stated inadequate milk secretion as the reason for starting complementary food early and 42 (47%) women stated lack of time to breastfeed due to work as the barrier. Two (2%) women stated vomiting by the child and breast condition of the mother as other barriers (Figure 3).

**Figure 4.**
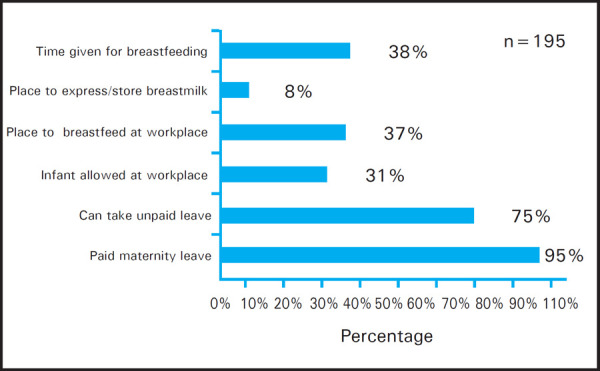
Maternity related facilities at workplace.

Total of 13 women had joined their service after six months of childbirth thus they were not asked about institutional provision related to breastfeeding. Out of 195 women, 185 (95%) women received paid maternity leave and only those women who were working since less than six months were devoid of it. Only 74 (38%) women said that they were given adequate time to breastfeed their child during working hour (Figure 4). Out of 76 women who were given time, 50 (68%) women said that they had to take permission from department or in charge and remaining 24 (32%) women arranged their working time with their colleagues in order to go to breastfeed their child.

## DISCUSSION

The results of this study indicate universal breastfeeding practice with more than half of the women initiating breastfeeding within one hour of childbirth which is consistent with the findings from study done by Polineni et al. and Ashoka et al. which found that 41% of the working mothers in Mysuru^12^ and 60% in Davengere, Karnataka initiated breastfeeding within one hour of childbirth.^13^ But it was very much low compared to findings from working mothers in North West Ghana (91%) which may be due to difference in study population as well as the mode of delivery i.e. 95.4% normal delivery in Ghana versus 29% normal delivery in our study.^14^ The mean duration of total breastfeeding was found to be very much lower than the findings from World Breastfeeding Trend^15^ but consistent with the study done among health workers in a tertiary level hospital of South India.^16^

It was found that pre-lacteal feeding was still present though very less compared to findings from study done by Polineni et al. in Mysuru which showed that 29% of the working women gave pre-lacteal feeding.^12^ The practice of pre-lacteal feeding in our study might have come less due to the mothers working in healthcare setting and being aware about the disadvantage of pre-lacteal feeding.

We found that excusive breastfeeding for any duration was higher but it decreased when reaching six months of age. Similar findings were seen in study done by Ashoka et al. in Karnataka,^13^ in which exclusive breastfeeding practice decreased from 100% at six weeks to 11% at six months. Dachew et al. and Amin et al. also found similar trend where exclusive breastfeeding at three months and six months were 49.4% and 35.9% in Ethiopia respectively^9^ and 54% and 12% in Malaysia respectively.^17^ Most of the women breastfeeding exclusively for three months may be related to the maternity leave duration and early rejoining of work. This study found work as the main reason for not breastfeeding the child exclusively which is similar to the findings from study done by Danso J in Kumasi Metropolis, Ghana, in which 90.5% of the working mothers stated work as the main barrier.^18^ Shah NZ also found in his study that most of the nurse mothers experienced work as the main barrier in Karachi.^19^

In our study, complementary feeding was also started before six months in almost half of the infants which is similar to the findings of study done in North West of Ghana (90%).^14^ The findings of our study was similar to results from study done by Ulak et al. in Bhaktapur in which 16% of the working mothers were found to introduce complementary food early.^20^

In our study paid maternity leave was received by majority of the women and additional unpaid leave could also be taken but supportive facilities for breastfeeding were very less. These findings are comparable to studies done by Boralingiah et al,^11^ Danso J,^18^ Dun-dery et al,^14^ Dachew et al,^9^ Amin et al,^17^ Shah NZ^19^ and Soomro et al.^21^ in which many women didn't have facilities at workplace to breastfeed, were not allowed to bring infants and were not given adequate breaks to breastfeed.

The study was conducted in tertiary level hospitals of urban area of Nepal thus this study may not represent women working in rural areas and other level hospitals. There might also be recall bias as breastfeeding practice of the mothers who had delivered in last five years was looked upon.

## CONCLUSIONS

Breastfeeding practice was universal but exclusive breastfeeding was lower and discontinued earlier with early initiation of complementary feeding. Lack of time due to work was the most common reason stated behind not exclusively breastfeeding and starting complementary food early. Thus, supportive environment should be provided to encourage women to exclusively breastfeed and continue breastfeeding for longer duration.
